# Detection of Ciprofloxacin in Urine through Sensitized Lanthanide Luminescence

**DOI:** 10.3390/s16122065

**Published:** 2016-12-05

**Authors:** Subhankar Singha, Kyo Han Ahn

**Affiliations:** Department of Chemistry, Pohang University of Science and Technology (POSTECH), 77 Cheongam-Ro, Nam-Gu, Pohang 37673, Korea; subhankar@postech.ac.kr

**Keywords:** ciprofloxacin, lanthanides, luminescence, urine

## Abstract

Ciprofloxacin, a fluoroquinolone antibiotic, is widely used for the treatment of bacterial infection in humans due to its broad antibacterial spectrum. An excessive use or overdose of ciprofloxacin on the other hand can cause several adverse effects not only to humans but also to microorganisms. Unabsorbed ciprofloxacin in the body is mostly excreted through urine and finally goes to the environment, providing a drug resistance pressure on bacteria. Hence a simple and efficient detection method of ciprofloxacin is necessary, which, for example, can be used to analyze ciprofloxacin content in urine. Although ciprofloxacin itself shows inherent fluorescence, direct fluorescent detection of ciprofloxacin in raw urine sample is difficult due to autofluorescence of urine by other components. Herein we report that a Tb(III) complex of DO3A (1,4,7,10-tetraazacyclododecane-1,4,7-triacetic acid) can be efficiently sensitized by ciprofloxacin to emit luminescence separately from the urine autofluorescence wavelength region. Tb-DO3A shows excellent sensitivity with a detection limit of three parts per billion in aqueous buffer solution. Further, Tb-DO3A is used to detect ciprofloxacin with high sensitivity and selectivity in a raw urine sample without any purification or separation procedures in the concentrations ranging from 1 µg·mL^−1^ to 50 µg·mL^−1^. The direct measurement of ciprofloxacin excreted in urine may be used to control overdose of the drug.

## 1. Introduction

Ciprofloxacin (Cipro), a third-generation synthetic fluoroquinolone antibiotic, is highly active against a broad spectrum of gram-negative and gram-positive bacteria. It is widely used for the treatment of infection caused by the bacteria that are resistant to other antibiotics including aminoglycosides and β-lactams [1]. The primary target of Cipro is topoisomerase II (a DNA gyrase), an essential bacterial enzyme that maintains super-helical twists in DNA [2]. The drug generally binds to the A subunit of the bacterial enzyme DNA gyrase and blocks DNA replication, which leads to rapid killing of bacteria through additional processes. Several side effects of Cipro as observed in humans include DNA damage [3], severe liver damage [4], and hematuria [5]. Also, Cipro is much less absorbed by the gastrointestinal tract in bioactive form than other fluoroquinolones [6]. Accordingly, the renal clearance of Cipro exceeds normal glomerular filtration rates, leading to a higher amount of renal tubular secretion to cause a relatively high Cipro concentration in urine [7]. A high proportion of patients under treatment with Cipro experiences adverse reactions in the gastrointestinal tract (nausea, vomiting), skin or the central nervous system. Such adverse effects and toxicities are occasionally worrisome in case of overdose or excessive use of the antibiotics [8]. Accordingly, determination of toxicities associated with higher doses and longer duration of therapy with Cipro and other fluoroquinolones needs additional studies.

At the same time, there is an increasing concern about bacterial resistance to antibiotics including Cipro, by means of mutations of altering the A subunit of DNA gyrase and other processes such as decreased drug permeation [9]. It is also observed that after oral administration, most of the antibiotics including Cipro are partially metabolized and mostly released via patient excreta into the municipal sewage system. If they are not eliminated during sewage treatment, the drugs that are released into the effluent enter the aquatic environment and eventually reach drinking water. If the concentrations of such antibiotics are high enough, effluent from hospitals, municipal sewage and sewage treatment plants may become a reservoir of bacteria resistance to antibiotics. A few studies showed that the amount of antibiotics emitted into hospital effluent is 10–20 times higher than the MIC_50_ (minimum inhibitory concentrations) of susceptible pathogenic bacteria (i.e., 2 µg·L^−1^) [10]. The amount of antibiotics also frequently exceeds the MIC_50_ even in municipal sewage and surface water. The presence of such a high amount of antibiotics in effluent and municipal sewage creates a resistance pressure on bacteria. Accordingly, it is necessary to analyze regularly the amount of drugs and active metabolites, especially the antibiotics adsorbed onto sewage sludge. Moreover, fluoroquinolones are also widely used as veterinary drugs in food-producing animals. To reduce the misuse of antibiotics, a regulation by the European Community has fixed a maximum residue limit (MRL) in edible animal products for some fluoroquinolones such as enorfloxacin and Cipro, which is settled at 30 µg·kg^−1^ for several edible animal tissues [11]. As the high risk of bacterial resistance could be a clinical issue, efficient analytical techniques are required to monitor residue levels of antibiotics such as Cipro in complex biological matrices.

Accordingly, several analytical methods have been reported for determining Cipro, which include high performance liquid chromatography (HPLC), high performance thin layer chromatography [12,13,14,15]. Widely used HPLC methods for the determination of Cipro or similar fluoroquinolone derivatives in biological material involve tedious extraction and clean-up steps prior to chromatography. As such, these methods are also not suitable for high throughput analysis for a pharmacokinetic study. The clinical significance of the potency and activities of Cipro in vivo depends on its pharmacology, efficacy, and toxicity, which again depend on the bioavailability or achievable serum level upon administration to body. Hence, there is a strong need to develop a new strategy towards the detection and quantification of antibiotics, which could be readily applied to biological fluid samples.

In this purpose, fluorescent detection could be an ideal means due to its high sensitivity and easy sample preparation procedures and non-invasive nature [16]. Cipro, being a representative quinolone carboxylic acid derivative, shows native fluorescence emission at 440 nm (Φ_F_ = 0.28) upon excitation at 278 nm in water [13]. Based on the native fluorescence of Cipro, a number of detection methods are reported such as spectrophotometry [17], spectrofluorimetry [18,19], and flow-injection with chemiluminescence detection [20,21]. In those existing methods a sample separation process is also necessary to remove proteins, amino acids and other biomolecules that interfere with the detection signal. Accordingly, those methods are not applicable to intact biological samples due to the high autofluorescence from the biological systems, the wavelengths of which overlap with that of Cipro (350–500 nm). To avoid such interference, it is necessary to develop a detection system that signals in the longer wavelength region. 

In recent years, use of lanthanide complexes (especially Eu^3+^ and Tb^3+^), specifically sensitized lanthanide luminescence, has been explored for the development of sensitive and selective detection methods for biological analytes [22]. The main advantages of this technique include emission at the longer wavelength, long luminescence life times, large Stokes shifts, and narrow emission bands, the properties of which can be used to alleviate the scattering interference and the background autofluorescence from the biomolecules. Cipro containing α-keto-acid functionality could chelate with lanthanide ions and act as an antenna to transfer its emission energy to the lanthanide ion. This sensitized emission with a large Stokes’ shift and narrow emission band in the longer wavelength region is expected to enable us to detect Cipro in biological samples without any sample preparation or pretreatment procedures. Nevertheless, only a few Ln^3+^-based fluorescent methods for Cipro detection are reported up to date, and those are often performed in conjunction with sample separating techniques [13,21,23,24]. There are two reports on the terbium-sensitized luminescence detection of ciprofloxacin or quinolone drugs, in which such sample extraction or separation procedures are not performed [25,26]. One of the methods used a mixture of TbCl_3_ and a surfactant (SDBS: sodium dodecylbenzene sulfonate) that enhanced terbium luminescence most effectively in its critical micelle concentration [25]. The analytical performance of such a system is thus dependent on the ionic strength of the medium. A background signal, that is, the Tb(III) emission, in the absence of a quinolone (norfloxacin) drug is also observed. The other method used a polymeric nanoparticle system generated from Tb(NO_3_)_3_·6H_2_O and adenine to detect Cipro in tablet and urine samples without complicated sample pretreatment processes, but the luminescence measurement was performed in the time-gated mode to avoid the autofluorescence/background signals from the samples [26]. For time-gated luminescence measurement a special fluorimeter that uses a nitrogen laser source is needed. Therefore, there is still a need to develop a convenient and reliable tool for the detection of quinolone antibiotics in intact biological samples.

Herein, we disclose a simple and direct detection method of Cipro in a biological fluid (urine) that does not require sample pretreatment or separation steps or a specialized fluorimeter, by further exploring the sensitized lanthanide luminescence by Cipro. The method could be used to detect the Cipro excreted in urine and hence to avoid the overdose of the drug. Also, the detection method could be applied to detect Cipro in plasma to monitor its appropriate dosage and to understand drug pharmacokinetics [27].

## 2. Results and Discussions

### 2.1. Ciprofloxacin Sensitized Lanthanide Luminescence

**Figure 1:**
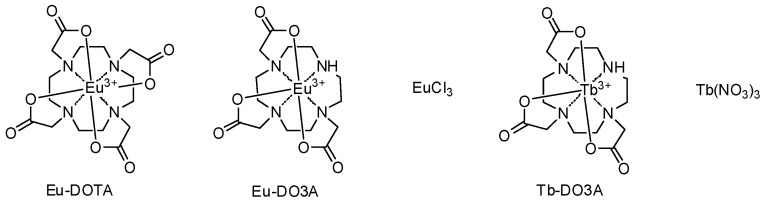
Structures of Eu^3+^ and Tb^3+^ complexes evaluated for the detection of ciprofloxacin (Cipro).

Among the lanthanides, europium ion (Eu^3+^) is well known for its characteristic red luminescence at 615 nm and 591 nm, corresponding to the ^5^D_0_ → ^7^F_2_ and ^5^D_0_ → ^7^F_1_ transitions, respectively. Due to the limitation of the direct excitation of Eu^3+^, a well-established phenomenon of using sensitized excitation of Eu^3+^ is possible by an appropriate organic ligand through energy transfer [28]. According to the Förster’s resonance energy transfer theory, the rate of energy transfer depends upon the extent of overlap of the emission spectra of the donor with the excitation spectra of the accepter and the distance between them [29]. At the same time as the energy transfer occurs from the triplet state of the sensitizer to the lanthanide, an organic dye ligand with high population of the triplet state could serve as an efficient sensitizer. Cipro has an intensely populated triplet state due to its relatively low energy gap between the S_1_ and T_1_ states (11.9 kcal·mol^−1^) and shows high phosphorescence/fluorescence intensity ratio (*P/F* = 2.92) [13]. So, a coordination complex of lanthanide ion with Cipro, upon excitation of the Cipro moiety, would show a sensitized luminescence through the Förster’s resonance energy transfer, where Cipro acts as energy donor and the lanthanide ion acts as energy acceptor. Keeping this in mind, we evaluated several Eu^3+^ complexes with different number of free co-ordination sites to find out the one that shows the largest sensitized luminescence change upon binding with Cipro. We chose Eu-DOTA [DOTA = 1,4,7,10-tetraazacyclododecane-1,4,7,10-tetraacetic acid], Eu-DO3A [DO3A = 1,4,7,10-tetraazacyclododecane-1,4,7-triacetic acid)], and EuCl_3_ as the probe candidates, which have the available co-ordination site number of one, two, and nine respectively for an analyte, as the maximum co-ordination number for Eu^3+^ is nine (Figure 1). 

**Figure 2:**
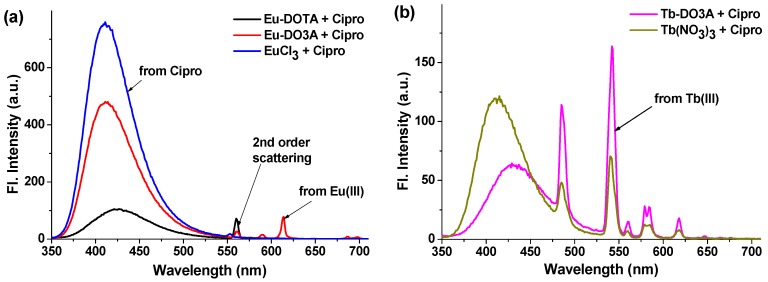
Emission behaviors of (**a**) Eu^3+^ and (**b**) Tb^3+^ complexes upon addition of Cipro. The emission spectra were collected immediately after addition of Cipro (10 µM) to a solution of each metal species (10 µM) in HEPES buffer (pH = 7.4), under excitation at 278 nm.

One equivalent of Cipro was added to each of metal complexes in HEPES buffer (pH = 7.4), and the luminescence from Eu^3+^ at 615 nm was monitored under excitation at 278 nm, a maximum absorption wavelength of Cipro. A characteristic emission band of Eu^3+^ was observed only in the case of Eu-DO3A among the three metal complexes examined (Figure 2a). It is thus concluded that Cipro coordinates effectively only to Eu-DO3A, which has two vacant co-ordination sites, and Cipro acts as the antenna molecule for the europium excitation. It is likely that the europium metal is bound with Cipro by the two vacant co-ordination sites of the keto and carboxylate groups. Upon binding with Cipro, the coordination sites of Eu^3+^ in Eu-DO3A are saturated and thus there are no available coordination sites for water molecules. Generally when a lanthanide ion forms a binary complex, its coordination sphere is usually filled by water molecules; such bound water molecules have a quenching effect on the lanthanide emission through vibrational relaxation [30].

**Figure 3:**
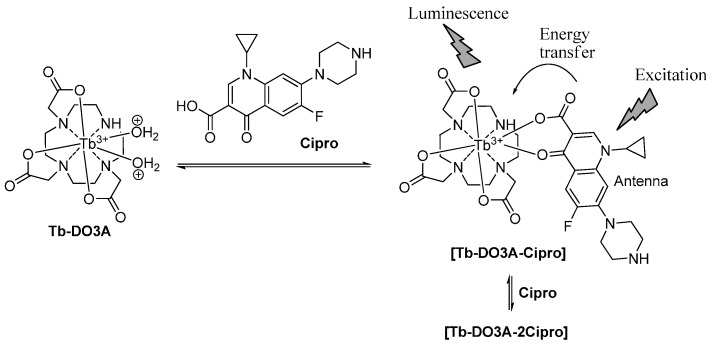
A sensing mechanism of Cipro with Tb-DO3A.

Although Eu-DO3A emits luminescence sensitized by the Cipro bound, its intensity was quite weak compared to that of the native fluorescence from Cipro (Figure 2a). The result suggested that the Förster’s resonance energy transfer from Cipro to europium is not efficient enough. The inefficient energy transfer could be rationalized by the lower extent of overlap between the emission spectra of the Cipro and the absorption spectra of the Eu^3+^, owing to the relatively larger energy gap between the triplet state of Cipro and the resonance level of Eu^3+^. On the contrary, a relatively smaller energy gap between the triplet energy level of Cipro and the resonance level of terbium (58.2 kcal·mol^−1^) than the case with europium would show an efficient energy transfer [31]. Accordingly, terbium (Tb^3+^) complex of DO3A (Tb-DO3A) was used as probe to observe lanthanide emission sensitized by Cipro. Upon addition of Cipro into a solution of Tb-DO3A, the four intense bands of terbium were observed, with a maximum peak at 542 nm (^5^D_4_ → ^7^F_5_ transition). Importantly, the native fluorescence of the Cipro molecule drastically decreased in the presence of Tb-DO3A, suggestive of an efficient Förster’s energy transfer from Cipro to Tb^3+^ (Figure 2b). It was observed that Tb(NO_3_)_3_ alone, upon chelation with Cipro, also shows the sensitized emission but with inferior performance: the terbium emission is lower while that of Cipro is higher than the case of Tb-DO3A (Figure 2b). These results show the significance of using the special ligand (DO3A) for Tb^3+^ in the sensitized detection of Cipro. Tb-DO3A has two vacant sites for coordination, which are used for binding Cipro. The resultant ternary complex, Cipro-bound Tb-DO3A, hence shows the best performance, showing enhanced sensitized emission from terbium and decreased emission from Cipro (Figure 3). Importantly, the sensitized emission band is fully separated from that of Cipro, which would enable us to detect Cipro by using a common fluorimeter, not by resorting to the time-gated luminescence measurement function available in advanced instruments. Accordingly, Tb-DO3A is further used for sensitive detection of Cipro in a biological sample such as urine.

### 2.2. Fluorescence Study with Tb-DO3A and Ciprofloxacin

**Figure 4:**
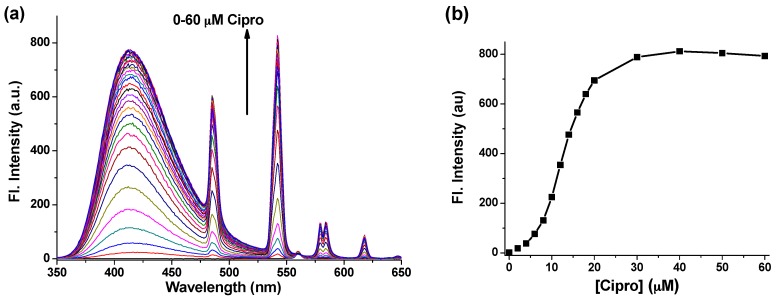
(**a**) Emission changes of Tb-DO3A (10 µM) with the increasing concentrations of Cipro (0–60 µM) in HEPES buffer (pH = 7.4); (**b**) Plot of terbium emission intensity at 542 nm vs. Cipro concentration. The spectra were recorded after excitation at 278 nm.

Given that Tb-DO3A emits the characteristic fluorescence sensitized by Cipro, first we assessed its sensing efficiency in HEPES buffer at pH = 7.4. Cipro was added with increasing concentration (0–60 µM) to a solution of Tb-DO3A at a fixed concentration (10 µM) in the HEPES buffer, and the emission spectrum of the resulting solution was collected under excitation at 278 nm. The fluorescence titration data given in Figure 4a shows a gradual increase of the terbium emission peaks (485, 542, 584, and 618 nm) with the maximum intensity at 542 nm. Interestingly, the emission intensity reached the maximum after addition of more than two equivalents of Cipro with respect to Tb-DO3A, suggestive of 1:2 stoichiometry between Tb-DO3A and Cipro (Figure 4b). This result also suggests that the second Cipro molecule can favorably compete with the two carboxylate ligands of DO3A to form a quaternary complex, [Tb-DO3A-2Cipro] (Figure 3). Such a 1:2 binding mode was also observed for other [Tb–Cipro] complexes [13]. Moreover, this stoichiometry is also similar to those reported for other triply charged Bi^3+^ and A1^3+^ complexes coordinated with norfloxacin, a fluoroquinolone drug [32]. Hence, to observe maximum luminescence signal from a fixed concentration of Tb-DO3A, we need around two equivalents of Cipro.

**Figure 5:**
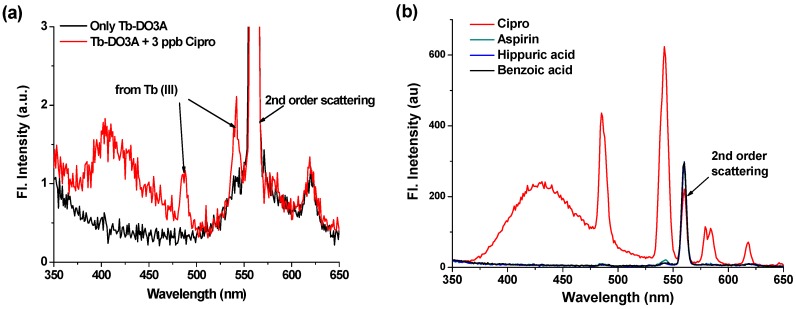
(**a**) Emission changes of Tb-DO3A (5 μM) upon addition of Cipro (3 ppb). The fluorescence data were obtained by excitation at 278 nm in HEPES buffer (pH = 7.4). This data shows a signal-to-background ratio of more than three. (**b**) Selective luminescence response of Tb-DO3A (10 µM) towards Cipro over other analytes possibly present in urine, such as aspirin, hippuric acid, and benzoic acid. Each of the analytes was added at a concentration of 20 µM in HEPES Buffer (pH = 7.4). The spectra were recorded under excitation at 278 nm.

To assess the sensitivity of Tb-DO3A, we measured the lower detection limit (LOD) of Cipro. Tb-DO3A showed excellent sensitivity towards Cipro in HEPES buffer even at the concentration of Cipro down to three parts per billion (9 pM) level, with the terbium emission signal (at 542 nm) to noise ratio of more than three (Figure 5a).

Our final goal is to develop a simple detection system of Cipro in intact biological samples, especially in urine samples. If we could measure the amount of excreted Cipro that was not absorbed in the body after taking the drug, it would be possible to measure the drug bioavailability, i.e., an absorbed amount of Cipro in the body (by excluding the amount of excreted Cipro from the amount of orally taken), which, in turn, could help us to control the dose of the drug and thus to avoid overdose.

Prior to detection of Cipro in urine, we evaluated the selectivity of Tb-DO3A toward potentially competing compounds remaining in urine. Among the normal compositions of urine [33], aspirin, hippuric acid, and benzoic acid could act as sensitizers upon coordination with lanthanide ions. Gratifyingly, Tb-DO3A shows excellent selectivity over these compounds, providing terbium luminescence only in presence of Cipro but not in other cases (Figure 5b).

### 2.3. Ciprofloxacin Detection in Urine Sample

**Figure 6:**
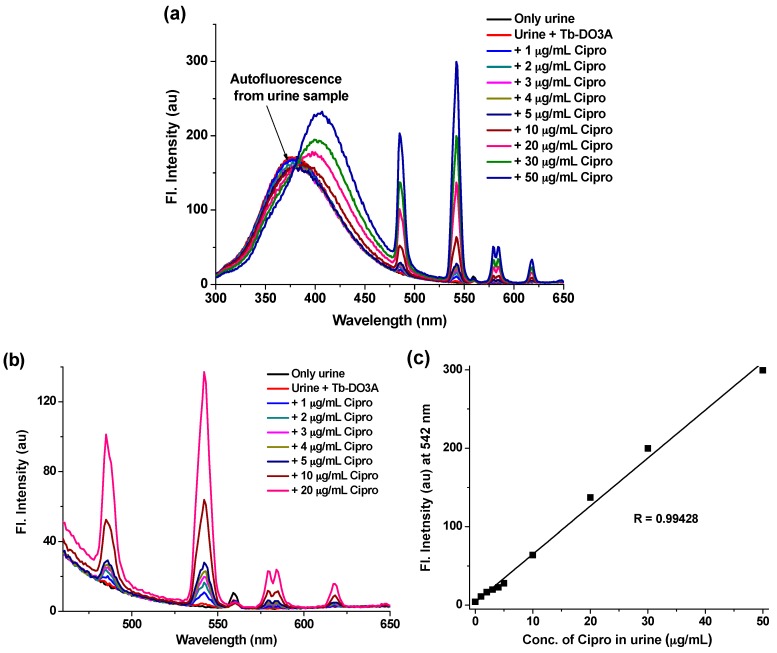
(**a**) Full emission spectra of urine, urine treated with Tb-DO3A, and urine treated both with Tb-DO3A and varying concentration of Cipro. The raw urine was diluted 10-times with DI-water. Tb-DO3A was added at a fixed concentration of 20 µM. The Cipro was added at various concentrations ranging from 1 µg·mL^−1^ to 50 µg·mL^−1^ with respect to the actual raw urine sample. The spectra were recorded after excitation at 278 nm. (**b**) The terbium emissions observed at the lower Cipro concentration region, which was produced from (a). (**c**) The linear intensity changes of terbium emission at 542 nm with increasing concentrations of Cipro, as measured in urine sample.

Finally, we applied Tb-DO3A for the detection of Cipro in intact urine. To this end, urine from a healthy volunteer was collected. The raw urine was diluted 10-times by adding DI water. Without any other pretreatment, the diluted urine was directly used for the fluorimetric assay. Tb-DO3A was added to a fixed volume of the diluted urine (2.5 mL) to make a final concentration of Tb-DO3A at 20 µM. Then Cipro was spiked to that solution at various concentrations, ranging from 1 µg·mL^−1^ to 50 µg·mL^−1^, with respect to the actual raw urine. It has been observed that urine itself shows strong autofluorescence in the wavelength region of 300–475 nm, which remains merged with the native fluorescence of Cipro (Figure 6a). This auto-fluorescence of urine samples is likely to be originated from the amino acids, proteins and biomolecules present in the raw urine sample [33]. To remove this interfering autofluorescence, most of the previous detection protocols based on Cipro’s native fluorescence were performed after laborious sample preparation steps to remove those biomolecules and proteins from urine [13,14,17,18,19,20,21,23,24]. Hence, those methods are not applicable to direct fluorimetric measurement for biological samples. On the contrary, we can conduct the Cipro detection without any sample preparation steps, as the sensitized luminescence of Tb-DO3A shows distinct emission peaks only in the presence of Cipro with little interference from other urine components (Figure 6a). Although the native fluorescence of Cipro was completely buried under the auto-fluorescence of urine, still the strong terbium luminescence peak at 542 nm (which is away from the urine auto-fluorescence region) was clearly observed upon treatment with Tb-DO3A even in the lower concentration range of Cipro (<10 mg·mL^−1^) (Figure 6b). The terbium luminescence increases linearly with the concentration of Cipro (Figure 6c), which would provide a convenient way to determine the unknown concentration of Cipro in urine samples. According to reported data, after oral administration of a Cipro tablet (250 mg) by a healthy human, the minimum amount of intact Cipro excreted through urine is ~4 µg·mL^−1^, which is dependent on the time and day of dose [27]. Our luminescence detection method using Tb-DO3A shows high sensitivity even in a raw urine sample; hence, it is suitable for the detection of such a low level of Cipro.

## 3. Experimental Section

### 3.1. Reagents and Solvents

Ciprofloxacin, europium chloride (EuCl_3_) and terbium nitrate (Tb(NO_3_)_3_) were purchased from Sigma-Aldrich Chemicals. Eu-(1,4,7,10-tetraazacyclododecane-1,4,7,10-tetraacetic acid) (Eu-DOTA) [34], Eu-(1,4,7,10-tetraazacyclododecane-1,4,7-triacetic acid) (Eu-DO3A) [35] and Tb-(1,4,7,10-tetraazacyclododecane-1,4,7-triacetic acid) (Tb-DO3A) [36] were prepared by following the literature procedures. All the solvents used were of analytical grade. Aqueous solutions were prepared using deionized water produced by a Milli-Q water system (Millipore, Darmstadt, Germany).

### 3.2. Apparatus and Instruments

All the apparatus used for synthesis of ligands and preparation of analytical solutions were made of Pyrex glass. A Photon Technical International Fluorescence system was used to record the emission spectra. HP Agilent 8453 spectrophotometer was used to measure the absorption spectra. All pH measurements were made with a pH meter of Thermo scientific, Orion 2 star pH benchtop.

### 3.3. Measurement Methods for the Detection of Ciprofloxacin

Solutions of the probes were prepared from the corresponding metal complexes by dissolving in DI water to make a stock solution at a concentration of 1 mM. The required amount of stock solution was added to 10 mM HEPES buffer (3 mL) of pH = 7.4 taken in a Quartz cuvette to prepare the probe solutions of 10 µM. Stock solutions of ciprofloxacin were prepared in DI water at a concentration of 0.5 mM. The required amount of ciprofloxacin stock solution was added gradually to the probe solution (3 mL) in HEPES buffer (pH = 7.4) in the quartz cuvette to make various final concentrations as needed. After each addition of ciprofloxacin, the emission spectra were recorded in the wavelength regions of 350–750 nm under excitation at 278 nm.

### 3.4. Sample Treatment for the Determination of Ciprofloxacin in Urine

Urine from a healthy volunteer was collected. The raw urine sample was diluted ten times with DI water. Without any other pretreatment, the diluted urine was directly used for the fluorimetric study. Tb-DO3A was added to a fixed volume (2.5 mL) of the diluted urine to make the final concentration of Tb-DO3A at 20 µM. A stock solution of Cipro (0.5 mM) in DI water was spiked to the solution at various concentrations, ranging from 1 µg·mL^−1^ to 50 µg·mL^−1^, with respect to the raw urine sample. After each addition, the emission spectra were measured upon excitation at 278 nm.

## 4. Conclusions

We have developed a simple yet efficient detection method for the antibiotic drug ciprofloxacin (Cipro) in urine sample based on the sensitized lanthanide luminescence. Evaluation of Förster’s resonance energy transfer efficiency between Cipro and several lanthanide complexes led to the terbium complex of DO3A (1,4,7,10-tetraazacyclododecane-1,4,7-triacetic acid) as a simple yet efficient detection system for Cipro. Non-luminescent Tb-DO3A emits characteristic terbium luminescence at 542 nm after binding to Cipro through the resonance energy transfer, enabling fluorescence detection of Cipro in the long wavelength region necessary to avoid the autofluorescence of proteins and biomolecules present in urine. The detection method is highly sensitive with a limit of detection down to three parts per billion level of Cipro in buffer solution. Also, the detection method is highly selective to Cipro over other potentially competing analytes in urine. Moreover, it is demonstrated that this simple detection method can be used to measure the amount of Cipro in a urine sample without any pretreatment or separation steps by using a common fluorimeter. The detection method could be applied to measure the bioavailability of Cipro and hence to control drug overdose and to study drug pharmacokinetics.
